# In-Package Atmospheric Cold Plasma Treatment and Storage Effects on Membrane Integrity, Oxidative Stress, and Esterase Activity of *Listeria monocytogenes*

**DOI:** 10.3390/microorganisms11030682

**Published:** 2023-03-07

**Authors:** Barun Yadav, M. S. Roopesh

**Affiliations:** Department of Agricultural, Food and Nutritional Science, University of Alberta, Edmonton, AB T6G 2P5, Canada

**Keywords:** cold plasma, storage, *Listeria*, ham, esterase activity, membrane permeabilization, oxidative stress

## Abstract

Atmospheric cold plasma (ACP) treatment can reduce bacterial pathogens in foods. Additional reduction in bacterial cells during storage after ACP treatment was previously reported. The underlying mechanisms of bacterial inactivation during ACP treatment and post-treatment storage need to be understood. This study investigated the changes in the morpho-physiological status of *Listeria monocytogenes* on ham surfaces after post-ACP-treatment storage of 1 h, 24 h, and 7 days at 4 °C. The membrane integrity, intracellular oxidative stress, and esterase activity of *L. monocytogenes* were evaluated by flow cytometry. *L. monocytogenes* cells were under high oxidative stress conditions with slightly permeabilized membranes after 1 h of post-ACP-treatment storage according to the flow cytometry data. During the extended storage of 24 h, the percentage of cells with a slightly permeabilized membrane increased; subsequently, the percentage of cells with intact membranes decreased. The percentage of *L. monocytogenes* cells with intact membranes decreased to <5% with a treatment time of 10 min and after 7 days of post-treatment storage. In addition, the percentage of *L. monocytogenes* cells under oxidation stress decreased to <1%, whereas the percentage of cells with completely permeabilized membranes increased to more than 90% for samples treated with ACP for 10 min and 7 days of post-treatment storage. With increased ACP treatment time, for 1 h stored samples, the percentage of cells with active esterase and slightly permeabilized membranes increased. However, during the extended post-treatment storage of 7 days, the percentage of cells with active esterase and slightly permeabilized membranes decreased to below 1%. At the same time, the percentage of cells with permeabilized membrane increased to more than 92% with an increase in ACP treatment time of 10 min. In conclusion, the higher inactivation after 24 h and 7 days post-ACP-treatment storage compared to 1 h stored samples correlated with the loss of esterase activity and membrane integrity of *L. monocytogenes* cells.

## 1. Introduction

*Listeria monocytogenes* is a foodborne pathogen that causes an estimated 178 cases of foodborne listeriosis annually in Canada [1]. Although listeriosis is a rare foodborne disease, it is linked to a disproportionately high level of hospitalization (>90%) and fatality (20–30%) [2,3]. Ready-to-eat (RTE) products, particularly meat and seafood, have been identified as “high risk” for *Listeria* contamination [4,5]. According to the Center for Disease Control and Prevention (CDC), the reported incidences of listeriosis remained unchanged between 2004 and 2014 [6], despite the strict guidelines [7]. *L. monocytogenes* is typically associated with RTE meat products as post-processing contamination from equipment surfaces such as slicers and conveyors belts [8,9].

The application of atmospheric cold plasma as a promising and innovative chemical-free antimicrobial technology to improve the microbial quality of various types of meats has been evaluated recently [10,11,12]. Cold plasma is an ionized gas composed of electrons, ions, charged particles, and free radicals. The ACP discharge generates cocktails of short- and long-lived reactive species including reactive oxygen species (ROS) and reactive nitrogen species (RNS), which have shown tremendous antimicrobial potential to injure and inactivate bacteria on food surfaces [13,14]. Previous studies reported inactivation of *L. monocytogenes* on RTE meat, including ham surface [15,16], beef jerky [17], fresh pork [18], and RTE meat bresaola [8]. Recent studies have indicated that ACP treatment can be used as a post-decontamination alternative for ready-to-eat meat products [8,13]. For example, Rød et al. [8] showed that in-package ACP treatment of RTE meat with MAP (30% O_2_ + 70% argon) gas resulted in a maximum 1.6 log reduction in *L. innocua* cell counts. In addition, some of these studies reported a significant increase in decontamination effects during post-treatment storage after in-package ACP treatments [19,20,21]. Han et al. [19] investigated the storage effects on the inactivation of *L. monocytogenes* cells suspended in PBS solution after in-package ACP treatment. They conducted in-package ACP treatment using air as the plasma-generating gas for 1 min. They reported 4.26 and 6.02 log CFU/mL reduction in *L. monocytogenes* after 1 h and 24 h post-treatment storage, respectively. In our previous study, we evaluated the effect of in-package ACP treatment on the reduction in *L. monocytogenes* at ham surface during post-treatment storage of 24 h and 7 days. The results showed an additional 3 log reduction in *L. monocytogenes* cells after 7 days of storage compared to 24 h for samples exposed to ACP for 10 min [20]. However, the underlying mechanisms of additional bacterial inactivation during post-ACP-treatment storage is not well-understood.

The plate counting method used for microbial inactivation efficacy evaluation does not consider the different physiological states of bacteria. Numerous studies have reported that the physical treatments, including ACP, can induce bacteria to exist in a viable but noncultural (VBNC) state or result in sublethal injury of bacteria [22,23]. Bacteria in VBNC or sublethally injured states are alive; however, the culture-based microbiological techniques can lead to underestimation of microbial cell number, which can survive the ACP treatment and post-treatment storage [22]. A better understanding of the physiological states of *L. monocytogenes* on the ham surface after in-package ACP treatments and the post-treatment storage is necessary to develop ACP-based decontamination processes and storage conditions.

Flow cytometry is an excellent tool for microbiological analysis, as it enables the evaluation of morphological and physiological changes in bacteria at a single-cell level. With the use of appropriate fluorescence dye, it is feasible to classify cells into three different categories: intact, metabolically active, and permeabilized cell mixtures [24,25]. Recently, the potential application of flow cytometry to assess the bacterial physiological status after ACP treatments was demonstrated [24,26,27,28]. In this study, the influence of post-ACP-treatment storage on membrane integrity, esterase activity, and oxidative stress of *L. monocytogenes* was investigated by flow cytometry.

## 2. Materials and Methods

### 2.1. Ham Sample Preparation

Ready-to-eat (RTE) cooked ham was prepared following the procedure described in Liu et al. [29]. Briefly, curing ingredients (wt/wt kg of lean meat) consisted of ice (16.71% of meat weight), sodium chloride (0.21% of meat weight), and sodium triphosphate (0.50% of meat weight); Prague powder (0.33% of meat weight (6% sodium nitrite, 94% NaCl)), sodium erythorbate (0.08%), and dextrose (2.45% of meat weight) were mixed in lean meat. The cooked ham was transferred on ice for 10 min and stored at 4 °C for overnight cooling. The cooled ham was sliced (2 mm thick, surface area 50 cm^2^) with a sterilized slicer, vacuum packed, and stored at 0 °C until use.

### 2.2. Bacterial Culture Preparation

The *L. monocytogenes* strain (FSL J1- 177) was streaked from −80 °C stock cultures on tryptic soy (TS) agar plates and incubated at 37 °C for 48 h to obtain single isolated colonies. The individual colonies were picked, transferred in 5 mL tryptic soya broth (TSB), and incubated in an orbital shaker at 37 °C (200 RPM) for 24 h. After overnight growth, 50 µL inoculum was transferred into 5 mL TS broth and incubated in an orbital shaker at 25 °C to closely mimic the processing environment temperature for 24 h. It was centrifuged twice at 5311× *g* for 2 min, and the supernatant was discarded. The cell pellets were resuspended in 1 mL of 0.85% NaCl to obtain a final cell count of approximately 8 log CFU/cm^2^ on the ham surface.

### 2.3. Inoculation and Packaging

The general inoculation and sample packaging procedures used in this study were described in [20]. In brief, vacuum-packed cooked ham was opened and cored to obtain samples with a surface area of 1 cm^2^. The top surface of the sample was spot inoculated with 10 µL of cells to reach final cell concentration of 10^8^ CFU/cm^2^, and the control samples were inoculated with the same volume of 0.85% NaCl. With a micropipette tip, the inoculum was spread on the ham surface and held in a biosafety cabinet for 10 min on a sterile tray. Using forceps, individual inoculated ham samples were aseptically transferred into 76.2 µm thick nylon and polypropylene-coextruded vacuum bags (Unipac Packaging Products Ltd., Edmonton, AB, Canada). The ham samples were packaged in modified atmosphere packages with 20% O_2_ + 40% N_2_ + 40% CO_2_ using a gas-flush packaging machine (Model C200; Multivac, Kansas City, MO, USA). Mixtures of gases with selected concentrations were selected based on our previous findings, i.e., 20% O_2_ + 40% N_2_ + 40% CO_2_ showed higher inactivation of *L. monocytogenes* on the ham surface [20]. The final gas volume per package averaged approximately 20 cm^3^.

### 2.4. Cold Plasma Treatment

The in-package ACP treatment system used in this study was described in [20]. A dielectric barrier discharge (DBD) (PG 100-3D, Advanced Plasma Solutions, Malvern, PA, USA) system under an atmospheric pressure condition with output voltage 0 to 30 kV at 3.5 kHz was used to generate plasma inside the package. The discharge gap between the high-voltage electrode (2.5 cm diameter) and ground electrode was equal to an in-package sample filled with gas (approx. 5 mm). The polyethylene bag and gas act as additional barriers between the ham and top electrode and the gap between the top high-voltage electrode and ham sample was approximately 3 mm. For in-package ACP treatment, individually packaged ham samples were placed within the plasma discharge region between two electrodes and treated for 1, 2.5, 5, and 10 min at 30 kV and 3.5 kHz under atmospheric temperature (21 ± 2 °C) and pressure. Prior to ACP treatment, the inoculated packaged ham samples were stored at 4 °C for 18 h for a logistic reason and to attain a uniform time interval between packaging and ACP treatments. All the experiments were performed in triplicate.

### 2.5. Total Viable Cell Counts

The surviving cell population of *L. monocytogenes* on the ham surface was analyzed after 1 h, 24 h, and 7 days of storage at 4 °C. The absence of background microorganisms was verified by plating un-inoculated packaged samples under the same conditions as the treated samples for each experiment. For microbial enumeration, both ACP-treated and untreated packaged samples were aseptically opened inside the biosafety cabinet and transferred into 50 mL falcon tubes containing 3 mL of 0.85% NaCl solution. Samples were vortexed for 60 s to detach cells from the ham sample prior to serial dilution. Aliquots of 0.1 mL of appropriate dilutions were plated on TSA to estimate total viable counts. Plates were incubated at 37 °C for 48 h for colony growth. Counts were converted to log_10_ CFU/cm^2^ for statistical analysis.

### 2.6. Flow Cytometry Analysis

The analysis was performed using a flow cytometer (BD LSR-Fotessa, BD Biosciences, San Jose, CA, USA), equipped with a 50 mW blue Ar laser emitting at a wavelength of 488 nm and a 50 mW yellow Ar laser emitting at a wavelength 561 nm to excite green (530 ± 30 nm) and red fluorescence (610 ± 15 nm), respectively. The green fluorescence of SYTO9, carboxyfluorescein (cFDA), and dichloro-dihydro fluorescein diacetate (H_2_DCFDA) was received in the Y530 photomultiplier with a bandpass filter of 530 ± 30 nm and the fluorescence of propidium iodide (PI) was acquired in the Y610 photomultiplier with a bandpass filter (610 ± 15 nm). The fluorescence signals were collected on logarithmic scales, and data were acquired in FACSDiva 8 software. FCS files were extracted from the FACSDiva 8 software, and obtained data were analyzed using FlowJo software (Tree Star, Ashland, OR, USA). For every stained bacterial cell, samples of at least 10,000 events were collected at a flow rate of approximately 250–500 events per sec. The cell density plots obtained by flow cytometry analysis were divided into four regions with respect to green and red fluorescence intensity. The regions were manually adjusted based on green and red fluorescence intensity to include more than 95% of cells from unstained controls as green- and red-fluorescence negative. The mean of the percentage values obtained from three independent density plots were calculated and presented on a bar graph, where the x-coordinate was the treatment time and the y-coordinate was the percentage of stained cells.

### 2.7. Membrane Integrity

SYTO9 and propidium iodide (PI) were used to differentiate between intact and damaged cell membranes. The stock solutions of 20 mM PI and 3.33 mM SYTO9 were procured (Thermo Fisher Scientific, Waltman, MA, USA) and stored at −20 °C. The cell-staining procedure was adapted from Berney et al. [30] with minor modifications. On the day of the experiment, a fresh working solution of SYTO9 (20 µM) and PI (60 µM) was prepared in 0.85% NaCl solution. The bacterial cell suspension was gently vortexed, and an aliquot of 25 µL of SYTO9 and PI was added into each tube to reach a final concentration of 8 µM SYTO9 and 20 µM PI and incubated in the dark for 15 min. Flow cytometry reading was collected no more than 30 min after incubation.

### 2.8. Esterase Activity and Membrane Permeabilization

The esterase activity of *L. monocytogenes* cells after post-ACP-treatment storage was assessed using a membrane-permeable dye, 5(6)-carboxyfluorescein diacetate mixed isomers (cFDA). This dye is nonfluorescent in its native form, but extremely intense fluorescence is produced upon hydrolysis by nonspecific intracellular esterase. cFDA does not stain dead cells. The stock solution of 5(6)-carboxyfluorescein diacetate mixed isomers (cFDA) (Thermo Fisher Scientific, Waltman, MA, USA) was dissolved in dimethyl sulfoxide to achieve a concentration of 1 mM. The bacterial cells from the ham surface were detached by vertexing for 60 s in 3 mL of 0.85% NaCl. The staining procedure for *L. monocytogenes* cells was adapted from Braschi et al. [31] and was adjusted with minor modifications. An aliquot of 150 µL of bacterial cell suspension was carefully pipetted into a 5 mL tube. The cell suspension was incubated in dark with 50 µM cFDA for 15 min at 37 °C with shaking. The physiological status of cells can be measured using membrane-impermeant PI. Therefore, 20 µM PI was added after 15 min of incubation with cFDA and stored in dark at room temperature for 10 min before flow cytometry measurements.

### 2.9. Oxidative Stress and Membrane Permeabilization

The stock solution of 6-carboxy-2′,7′-dichlorodihydrofluorescin diacetate (H_2_DCFDA), a membrane-permeable fluorescence dye (Thermo Fisher Scientific, Waltman, MA, USA), was dissolved in dimethyl sulfoxide to achieve a concentration of 1 mM. An aliquot of 150 µL of bacterial cell suspension was carefully pipetted into a 5 mL tube. To assess the intracellular oxidative stress level of *L. monocytogenes*, the cell suspension was incubated with 20 µM H_2_DCFDA for 15 min at 37 °C with shaking, following the procedure described in Carré et al. [27] with slight modifications. To measure the bacterial cell membrane permeabilization along with oxidative stress, membrane-impermeant dye PI was added with a final concentration of 20 µM after 15 min of incubation with H_2_DCFDA. The tube containing the bacterial cell suspension and dye was gently vortexed and stored in dark at room temperature for an additional 10 min before flow cytometry measurements.

### 2.10. Statistical Analysis

Data on the response variables were subjected to analysis of variance using a generalized linear mixed-effects procedure of the SAS^®^ University edition (Proc Glmmix; SAS studio 3.71, Cary, NC, USA). The treatment factors, ACP treatment time, and post-ACP storage time were used as fixed effects. The level of significance reported in the test was *p*-value ≤ 0.05 using Tukey’s honest test.

## 3. Results

### 3.1. Effect of Post-ACP-Treatment Storage Time on Total Viable Cell Counts

**Figure 1:**
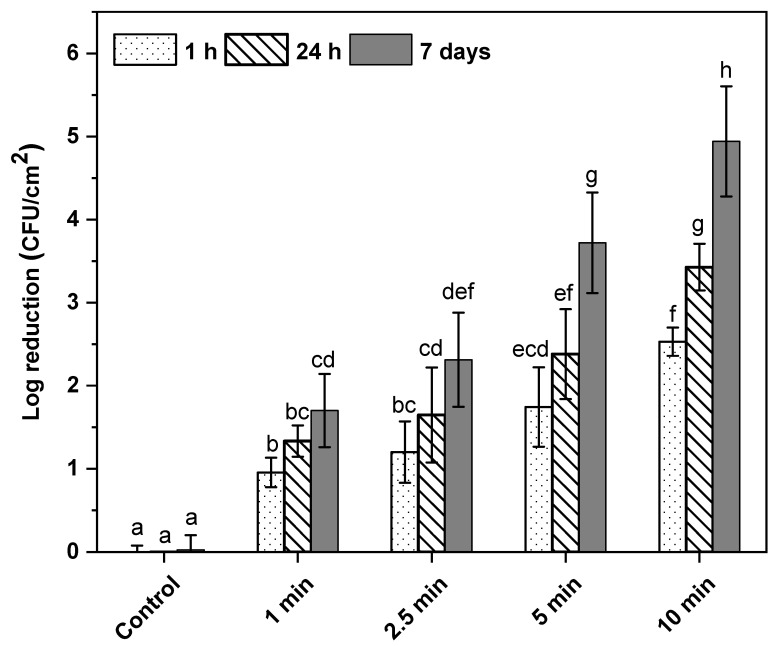
Mean cell counts of *L. monocytogenes* on ham surface after in-package ACP treatment and storage. White bar with dots represents post-treatment storage for 1 h at 4 °C; white bar with hatch lines represents post-treatment storage for 24 h at 4 °C; grey bar represents post-treatment storage for 7 days at 4 °C. Data are shown as means ± standard deviations (*n* = 3). Treatment means with different letters are significantly different.

The inactivation levels of *L. monocytogenes* on the ACP-treated samples after post-treatment storage of 1 h, 24 h, and 7 days at 4 °C were determined. The initial cell count of *L. monocytogenes* on the ham surface was ~8 log CFU/cm^2^. The results indicated a significant decrease in *L. monocytogenes* cell counts compared to untreated control at all tested treatment times after 1 h, 24 h, and 7 days post-treatment storage at 4 °C (Figure 1). Both the ACP treatment time and the post-treatment storage time had significant influence on the reduction in *L. monocytogenes* on the ham surface (Figure 1). An increase in treatment time from 1 min to 2.5 min did not significantly reduce the cell counts, but an additional 2.5 min treatment resulted in a significant reduction in the cell counts of *L. monocytogens* (Figure 1). Maximum log reductions of 2.5, 3.5, and 4.9 log CFU/cm^2^ in *L. monocytogenes* cell counts were observed for samples exposed to 10 min of ACP treatment, followed by 1 h, 24 h, and 7 days’ post-treatment storage, respectively. Statistical analysis revealed that the interaction effect between ACP treatment time and post-treatment storage time was significant, which means that the reduction in *L. monocytogenes* depended on ACP treatment time and post-treatment storage time and vice versa. The 2.5 min of ACP treatment, followed by 24 h and 7 days’ post-treatment storage, resulted in 1.6 and 2.3 log CFU/cm^2^ reductions in the *L. monocytogenes* population, respectively, whereas 5 min ACP treatment, followed by 24 h and 7 days post-treatment storage, resulted in a significantly higher reduction in *L. monocytogenes* cell counts, with log reductions of 2.3 and 3.7 log CFU/cm^2^, respectively. A significantly higher reduction in cell counts of *L. monocytogenes* was observed for samples stored for 7 days, followed by 24 h and 1 h, respectively, irrespective of ACP treatment time.

### 3.2. Effect of Post-ACP-Treatment Storage Time on Membrane Integrity

**Figure 2:**
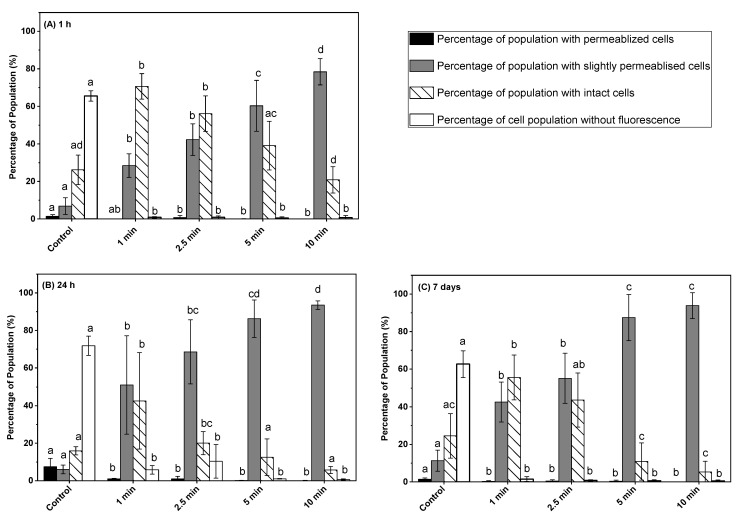
Membrane integrity measurements of *L. monocytogenes* cells on ham surface using SYTO9 and PI in combination after in-package ACP treatment storage at 4 °C for (**A**) 1 h, (**B**) 24 h, (**C**) 7 days. Black bar represents permeabilized cells (PI- fluorescence); grey bar represents slightly permeabilized cells (SYTO9 + PI fluorescence); white bar with hatch lines represents intact cells (SYTO 9 fluorescence); white bar represents cells without fluorescence. Data are shown as means ± standard deviations (*n* = 3). Treatment means with different letters are significantly different.

The distribution of stained *L. monocytogenes* cells varied based on the ACP treatment time and post-treatment storage time (Figure 2A–C). The percentage of unstained (SYTO9 + PI negative) cells of untreated control samples remains greater than 62% after 1 h, 24 h, and 7 days of post-treatment storage at 4 °C (Figure 2A–C). ACP treatment for 1 min, followed by 1 h, 24 h, and 7 days’ post-treatment storage of *L. monocytogenes* on the ham surface, reduced the unstained cell population to 0.8, 5.8, and 1.5%, respectively. The percentage of *L. monocytogenes* with intact cell membranes increased from 26% (untreated controls) to 70% after 1 min of ACP treatment, followed by 1 h of post-treatment storage at 4 °C. During post-treatment storage of 24 h, the percentage of cells with intact membranes decreased significantly from 70 to 42%; it was increased again to 55% after 7 days’ post-treatment storage. Irrespective of post-treatment storage time, an increase in ACP treatment time from 1 to 2.5 min did not significantly change the percentage of cells with intact membranes. A significant decrease in *L. monocytogenes* cells with intact membranes was observed after 5 and 10 min of ACP treatments for all the post-treatment storage times (Figure 2A–C). In fact, the percentage of *L. monocytogenes* with intact cell membranes remained almost below 6% after 10 min of ACP treatment followed by 24 h and 7 days of post-treatment storage. The percentage of *L. monocytogenes* with slightly permeabilized membranes (SYTO9 + PI stained) was significantly greater for all the ACP treatment times and post-treatment storage times compared to untreated controls. Regardless of post-treatment storage time, no significant difference in the percentage of cells with a slightly permeabilized membrane was observed after 2.5 min of ACP treatment compared to 1 min of ACP treatment. A significant increase in the percentage of *L. monocytogenes* with the slightly permeabilized cell membrane was observed after 5 min of ACP treatment compared to 2.5 min for all post-treatment storage times (Figure 2A–C). The ACP exposure of 10 min to *L. monocytogenes* on the ham surface followed by 1 h, 24 h, and 7 days’ post-treatment storage significantly increased the percentage of cells with slightly permeabilized membranes to 78, 93, and 93%, respectively. Irrespective of ACP treatment time and post-treatment storage time, the percentages of *L. monocytogenes* with permeabilized cell membranes (PI stained) were lower than 1% for all treated samples.

### 3.3. Effect of Post-ACP Treatment Storage Time on Esterase Activity

**Figure 3:**
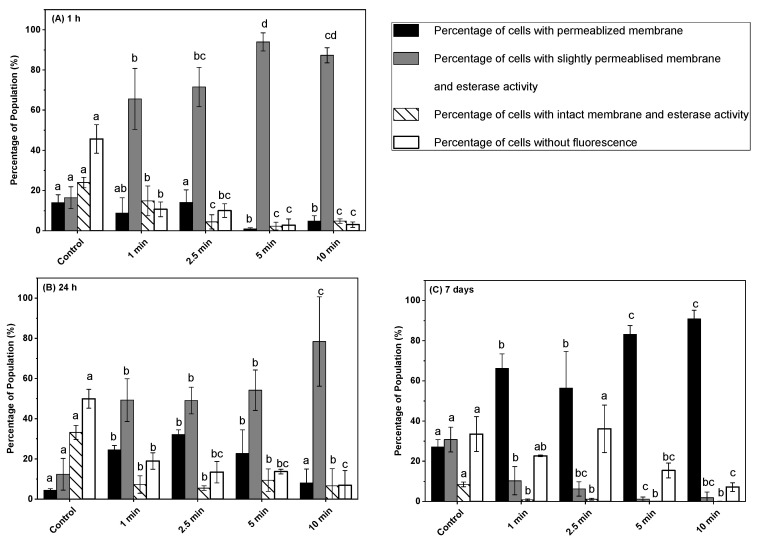
Esterase activity and membrane integrity measurements of *L. monocytogenes* cells on ham surfaces using cFDA and PI in combination after in-package ACP treatment storage at 4 °C for (**A**) 1 h, (**B**) 24 h, (**C**) 7 days. Black bar represents permeabilized cells (PI- fluorescence); grey bar represents slightly permeabilized cells with esterase activity (cFDA + PI fluorescence); white bar with hatch lines represents intact cells with esterase activity (cFDA fluorescence); white bar represents cells without fluorescence. Data are shown as means ± standard deviations (*n* = 3). Treatment means with different letters are significantly different.

The impact of ACP on intracellular compounds was investigated by measuring esterase activity of *L. monocytogenes* cells after ACP treatment, followed by post-treatment storage of 1 h, 24 h, and 7 days using cFDA and PI in combination (Figure 3A–C). The action on membrane integrity, measured by SYTO9 and PI in combination, dependent on ACP treatment time and post-treatment storage time was confirmed by measuring esterase activity. The percentage distribution of unstained (cFDA+ PI negative), cFDA-stained, cFDA + PI-stained, and PI-stained cells in untreated control samples were 45.6, 24, 16.4, and 1.9%, respectively. After 1 min of ACP treatment followed by 1 h post-treatment storage time, the percentage of unstained and intact *L. monocytogenes* with esterase activity was decreased significantly from 45.6 to 10.6 and from 24 to 14.8%, respectively, compared to untreated control. The percentage of cells with slightly permeabilized membrane and esterase activity was increased at the same time from 16.4 to 65.6%. The number of slightly permeabilized membranes of *L. monocytogenes* cells with esterase activity increased to the highest level of 93.9% after 5 min of ACP treatment followed by 1 h of storage, whereas all other fractions of the population were less than 3% (Figure 3A). During the extended duration of post-treatment storage for 24 h and 7 days, a significant increase in the percentage of permeabilized cells was observed compared to 1 h stored samples, whereas the percentage of slightly permeabilized cells with esterase activity decreased significantly for all tested treatment times (Figure 3A–C). The permeabilized cell population during 1 h to 7 days of post-treatment storage increased with the increase in ACP treatment time and reached 90.9% for samples treated with 10 min of ACP treatment, while the percentage of slightly permeabilized cells with esterase activity decreased at the same time from 87.3 to 1.9%.

### 3.4. Effect of Post-ACP Treatment Storage Time on Oxidative Stress

**Figure 4:**
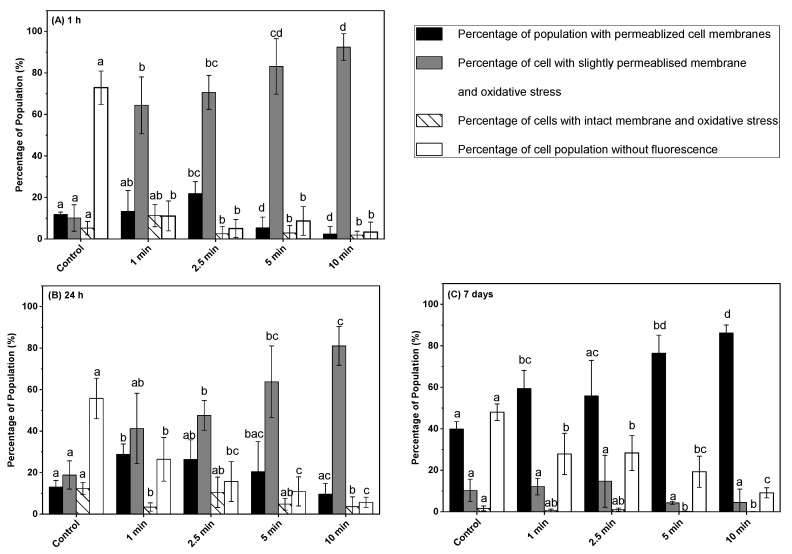
Oxidative stress and membrane integrity measurements of *L. monocytogenes* cells on ham surface using H_2_DCFDA and PI in combination after in-package ACP treatment storage at 4 °C for (**A**) 1 h, (**B**) 24 h, (**C**) 7 days. The black bar represents permeabilized cells (PI- fluorescence); grey bar represents slightly permeabilized cells with oxidative stress (H_2_DCFDA + PI fluorescence); white bar with hatch lines represents intact cells with oxidative stress (H_2_DCFDA fluorescence); white bar represents cells without fluorescence. Data are shown as means ± standard deviations (*n* = 3). Treatment means with different letters are significantly different.

The impact of ACP treatment followed by post-treatment storage of 1 h, 24 h, and 7 days on intracellular oxidative stress was investigated using H_2_DCFDA in combination with PI (Figure 4A–C). The percentage of unstained (H_2_DCFDA + PI negative) cells of untreated control samples significantly decreased from 72 to 11% after 1 min of ACP treatment following 1 h of post-treatment storage. At the same time, the percentage of cells with a slightly permeabilized membrane and oxidative stress increased significantly from 10 (control) to 64%. An increase in treatment time from 1 to 2.5 min followed by 1 h post-treatment storage time did not significantly change the percentage of cells with intact membranes and oxidative stress, completely permeabilized, and a slightly permeabilized membrane with oxidative stress. During the post-treatment storage of 24 h and 7 days, a slight increase in the percentage of unstained and permeabilized cells was observed compared to 1 h stored samples, while the percentage of cells with a slightly permeabilized membrane and oxidative stress decreased (Figure 4A–C). Samples treated with ACP for 10 min followed by 1 h storage showed 92% of slightly permeabilized cells with oxidative stress, whereas 24 h and 7 days’ post-treatment storage decreased it to 81% and 4%, respectively. However, the percentage of cells with permeabilized membrane without oxidative stress increased after 24 h and 7 days of post-treatment storage compared to 1 h stored samples, for all treatment times. For instance, samples exposed to 5 min of ACP followed by 1 h, 24 h, and, 7 days of post-treatment storage resulted in 5.3, 20.4, and 86% of cells with permeabilized membrane, respectively. Similarly, irrespective of ACP treatment time, a slight increase in the percentage of unstained cells was observed after 24 h and 7 days of post-treatment storage compared to 1 h post-treatment stored samples (Figure 4A–C). The percentage of intact cells with oxidative stress remained lower than 10% for all the tested treatment times and it further reduced during the post-treatment storage to lower than 1%.

## 4. Discussion

ACP discharges into atmospheric air or into packages filled with nitrogen and oxygen produce various reactive oxygen and nitrogen species (RONS), including free-radical superoxides and long-lived species including ozone, hydrogen peroxide, nitrates, and nitrites, that participate in microbial inactivation [32]. Among the generated plasma-reactive species, ozone, hydrogen peroxide, and nitrites are mainly responsible for the microbial inactivation [33,34]. The concentration of these major reactive species in ACP discharge is greatly dependent on the ACP treatment time, temperature, and gas composition inside the packages [20,35]. Plasma discharge in a process-gas mixture containing oxygen produces highly reactive oxygen species such as ozone (O_3_), hydrogen peroxide, nitrous gases, singlet oxygen, superoxides, and hydroxyl radicals. For instance, the presence of oxygen enhances the production of long-lived ozone (O_3_), hydrogen peroxide, and reactive nitrogen species (RNS) during the ACP treatment. The production of RNS was reported in the plasma discharge with air as the gas [36]. The combined effect of ROS with RNS can result in the enhanced antibacterial effect of ACP in comparison to either group alone [37]. The results of this study demonstrate that the antimicrobial efficacy of in-package ACP discharge on a ham surface significantly depended on the ACP treatment time and post-treatment storage. The treatment-time-dependent *L. monocytogens* inactivation on ham during post-treatment storage in this study was consistent with prior reports [8,19]. Han et al. [19] used a cell-recovery model to establish a relationship between ACP treatment time and post-treatment storage. They reported a treatment-time-dependent response of cell recovery during post-ACP-treatment storage. A similar inactivation effect was observed in this study, where ACP treatment of 10 min followed by 24 h and 7 days of storage showed a significantly higher reduction in *L. monocytogenes* compared to samples exposed to 5 min ACP followed by 24 h and 7 days of storage (Figure 1).

Although the enhanced antibacterial effect of post-ACP-treatment storage is well-documented, only limited information is available about their mechanisms of action on *L. monocytogenes* during post-treatment storage [19,21,28]. The membrane, cell wall, and intracellular component damages are generally considered as the main reasons for microbial inactivation [38]. Therefore, flow cytometry analysis was performed in this study to investigate the underlying inactivation mechanisms of *L. monocytogenes* after post-ACP-treatment storage of 1 h, 24 h, and 7 days at 4 °C. In this regard, membrane integrity, intracellular oxidative stress, and esterase activity of *L. monocytogenes* were measured by flow cytometry. The *L. monocytogenes* membrane integrity data showed a specific response trend for the ACP treatment time and post-treatment storage. Regarding membrane permeabilization, an increase in the percentage of slightly permeabilized cells and a decrease in the intact cell population after 24 h storage compared to 1 h storage indicate additional membrane damage during storage. This increase in membrane permeabilization during storage is likely due to the membrane oxidation caused by long-lived plasma-reactive species, particularly hydrogen peroxide. Previously, the high concentrations of hydrogen peroxide after 6 h and 18 h of post-treatment storage were reported [39]. Hydrogen peroxide is considered as one of the major contributors to bacterial inactivation resulting in cell membrane oxidation during ACP treatment [33]. Similar to these findings, a significant increase in the percentage of cells with a slightly permeabilized membrane after 24 h post-treatment storage was reported for *Citrobacter freundii* in apple juice treated with an ACP jet for 8 min [28]. The results of the 1 min ACP-treated sample showed that after 1 h, 24 h, and 7 days of storage, the percentage of *L. monocytogenes* cells with intact membranes varied. In contrast, a similar variation was not observed in samples exposed to extended ACP treatment times (5 min or 10 min) (Figure 2). In fact, the percentage of cells with intact membranes decreased significantly after 5 or 10 min of treatment followed by 1 h post-treatment storage and decreased further after 24 h and 7 days’ storage (Figure 2). These results and trends suggest that ACP treatment causes significant damage to the cell membrane of *L. monocytogenes*, and longer treatment times result in more pronounced damage, which becomes increasingly evident during post-treatment storage. Conversely, for samples exposed to the ACP treatment for only 1 min, the damage to the *L. monocytogenes* cell membrane was reversible, leading to some recovery of damaged cells during post-treatment storage and resulting in variations in the percentage of cells with intact membranes. No significant change in the percentage of slightly permeabilized cell population with an increase in post-treatment storage time from 24 h to 7 days was observed for samples exposed to ACP treatment for 5 or 10 min, indicating irreversible damage to the cells that led to the loss of cell recovery during long-term storage. Although the percentage of a slightly permeabilized cell population was comparable for samples after 24 h and 7 days’ storage, a significant decrease in the number of *L. monocytogenes* cells was observed after 7 days’ storage compared to 24 h storage. This implies that during long-term post-treatment storage, the permeabilization of cell membranes is one of the major causes of the loss of culturability, and a large fraction of permeabilized cells lost their ability to grow on a nutrient agar plate.

The gold-standard plate-counting method for inactivation efficacy evaluation does not consider the different physiological states of bacteria. The ACP treatment can induce bacteria to enter the viable but non-culturable (VBNC) state that can lead to underestimation of microbial cell numbers [22,23]. Recently, a few studies reported the use of cFDA in combination with PI to identify metabolically active (active esterase) and damaged bacterial cell populations after ACP treatment by flow cytometry [24,27,28]. In this study, the esterase activity of *L. monocytogenes* cells increased in response to ACP treatment, and the maximum value was found for 10 min ACP-exposed samples after 1 h storage. Similar to these findings, a treatment-time-dependent increase in the esterase activity of *Citrobacter freundii* in apple juice in response to ACP treatment was reported immediately after ACP treatment and after 3 h post-treatment storage [28]. A significant decrease in the esterase activity and increase in the percentage of permeabilized cells of *L. monocytogens* during subsequent post-treatment storage of 24 h and 7 days compared to 1 h storage suggests a loss of metabolic activity. This result suggests that after ACP treatment, a large fraction of *L. monocytogens* cells was metabolically active during 1 h of post-treatment storage. This finding is further supported with the observed higher colony-forming units of *L. monocytogens* on TSA plates in 1 h stored samples compared to 24 h and 7 days.

ACP discharge in the presence of nitrogen, oxygen, and carbon dioxide generates various short-lived and long-lived reactive oxygen and reactive nitrogen species [32,38]. These reactive species can inactivate bacteria through cell membrane disruption via membrane disintegration and generation of intracellular ROS species [38,40]. In this study, the intracellular oxidative stress of *L. monocytogenes* cells after post-treatment storage was analyzed using dye exclusion methods with H_2_DCFDA and the PI assays by flow cytometry. The PI stains the bacterial DNA when the cell membranes are permeabilized, and H_2_DCFDA reveals the oxidative stress of bacteria. The results from these combined assays revealed that a higher percentage of the cell population was under a high oxidative stress environment with slightly permeabilized cell membranes in response to ACP treatments after 1 h storage. An increase in the percentage of cells with a slightly permeabilized membrane and oxidative stress after 24 h storage indicated that an interactive effect of ACP generated long-lived reactive species with the bacterial cell membranes during storage. Previous studies reported the presence of a traceable amount of hydrogen peroxide, ozone, and nitrous compounds inside the package after post-treatment storage [19,39]. Apart from ACP-generated reactive species, oxidatively stressed bacteria generate several peroxides and superoxides inside cells that cause a series of oxidation-reduction reactions and further generate a large amount of short-lived hydroxyl radical species, which consequently can cause intracellular damage [33]. The higher percentage of permeabilized cell population without oxidative stress (only PI stained) after 7 days of storage compared to 1 h or 24 h suggests that the bacterial cells were no longer under oxidative stress conditions and lost their membrane integrity. This finding was further supported by the lack of culturability and loss of esterase activity in *L. monocytogens* cells after an extended duration of post-treatment storage.

## 5. Conclusions

This research studied the possible mechanisms of *Listeria monocytogenes* inactivation on a ham surface after post-ACP-treatment storage. A treatment-time-dependent decrease in the culturable *L. monocytogenes* population was observed during post-ACP-treatment storage. The flow cytometry method highlighted the physiological states of bacteria sub-population distribution, depending on the ACP treatment time and post-treatment storage. Flow cytometry data suggest that a very high percentage of *L. monocytogenes* cells were under high oxidative stress conditions with active esterase and slightly permeabilized membranes, even after 24 h of post-ACP-treatment storage. The data obtained from the flow cytometry analysis with active esterase activity and slightly permeabilized membrane immediately after 1 h of post-ACP-treatment storage, when compared to 24 h or 7 days, indicate that *L. monocytogenes* cells were metabolically active. This observation was confirmed by a higher colony-forming unit count on the TSA plate. However, the increase in the percentage of cells with permeabilized membranes and the decrease in esterase activity suggested additional oxidative damage to the *L. monocytogenes* cell membranes after 7 days of post-ACP-treatment storage at 4 °C. Both the flow cytometric data on esterase activity and plate-count method revealed a treatment-time-dependent decrease in the total active bacterial population after 7 days of post-treatment storage. Overall, the study results provide insight into the inactivation of bacteria during post-ACP-treatment storage and highlight the significance of storage in achieving greater bacterial inactivation. These findings are relevant to maintaining the microbial quality of ready-to-eat ham. Nevertheless, further research is needed to assess the effects of prolonged storage following in-package ACP treatment on the nutritional quality of RTE ham and the possibility of producing any toxic byproducts.

## Data Availability

Data is available on request.
